# A Comparison of Magnetic Resonance Angiography Techniques for the Evaluation of Intracranial Aneurysms Treated With Stent-assisted Coil Embolization

**DOI:** 10.7759/cureus.909

**Published:** 2016-12-02

**Authors:** Krishnamoorthy Thamburaj, Kevin Cockroft, Amit K Agarwal, Shyam Sabat, Paul Kalapos

**Affiliations:** 1 Neuroradiology, Penn State Hershey Medical Center; 2 Department of Neurosurgery, Penn State Hershey Medical Center; 3 Department of Radiology, Penn State Hershey Medical Center

**Keywords:** magnetic resonance angiography, intracranial stents, cerebral aneurysm, coil embolization, mra

## Abstract

**Aim:**

To identify the effective magnetic resonance angiography (MRA) technique to monitor intracranial aneurysms treated with stent-assisted coiling.

**Materials and Methods:**

Retrospective analysis of various MRA techniques was performed in 42 patients. Three neuroradiologists independently compared non-contrast time of flight (ncTOF) MRA of the head, contrast-enhanced time of flight (cTOF) MRA of the head and dynamic contrast-enhanced MRA (CEMRA) of the head and neck or of the head. Digital subtraction angiography (DSA) was available for comparison in 32 cases. Inter-rater agreement (kappa statistic) was assessed.

**Results:**

Artifactual in-stent severe stenosis or flow gap was identified by ncTOF MRA in 23 of 42 cases (55%) and by cTOF MRA in 23 of 38 cases (60%). DSA excluded in-stent stenosis or occlusion in all 32 cases. No difference was noted between ncTOF and cTOF in the demonstration of neck remnants or residual aneurysms in three cases each. CEMRA of the head and neck or of the head was rated superior to ncTOF and cTOF MRA by all three investigators in seven out of eight cases. In one case, all three techniques demonstrated signifcant artifacts due to double stent placement during coiling. The kappa statistic revealed 0.8 agreement between investigators.

**Conclusions:**

In the assessment of stent-assisted coiling of intracranial aneurysm, both ncTOF and cTOF MRA show similar results. CEMRA tends to show better flow signals in stent and residual aneurysm.

## Introduction

Approximately 20% of detachable coil embolization of intracranial aneurysms may result in recurrence. It is essential that coiled aneurysms are monitored at regular intervals to identify residual or recurrent aneurysms [1].Assessment of the durability of the coil occlusion is typically done with MRA. Initially ncTOF MRA was used to assess coiled aneurysms [2-6]. Later, a cTOF technique was tested to improve the pitfalls associated with ncTOF. A few studies have reported an advantage for cTOF over ncTOF [7]. However, many studies do not see a benefit for cTOF over ncTOF [8-10]. Later, CEMRA was introduced to assess coiled intracranial aneurysms [7]. Some studies highlight the advantages of CEMRA over TOF. Others report a lack of advantage for CEMRA over TOF [10-11]. Further, some studies report the superiority of TOF over CEMRA [12]. Hence, some investigators advocate a combination of CEMRA and TOF in the assessment of coiled intracranial aneurysms [13]. In a recent meta-analysis, Kwee et al. [9] reported no statistically significant difference in the pooled sensitivity and specificity between ncTOF and contrast-enhanced techniques including CEMRA and cTOF. This situation is further complicated by the use of stent-supported embolization for aneurysms with a wide neck or complex geometry [14-16]. There are few studies on MRA assessment of stent-assisted coil embolization, and there is no clear consensus yet as to the most suitable technique [4,16-23]. In this study, we report our experience in the assessment of stent- assisted coil embolization with three main MRA techniques including ncTOF, cTOF and CEMRA.

## Materials and methods

Prior Institutional Review Board permission was obtained from Penn State Hershey Medical Center (approval number 33974EP) for this retrospective study. The study is HIPPA compliant. Patient informed consent was obtained.

A search of our neurointerventional database identified 91 cases of stent-assisted coiling of intracranial aneurysms from February 2005 to October 2009. Forty-nine cases were excluded from the study due to lack of either cTOF or CEMRA. Forty-two cases were available for final analysis, and all of them underwent ncTOF. Thirty-eight cases underwent cTOF and eight cases had CEMRA of the head or of the head and neck. MRA was performed with either 1.5T or 3T machines including Achieva (Philips Healthcare, Andover, MA), Magnetom Avanto (Siemens Healthineers, Erlangen, Germany) and Espree (Siemens Healthineers, Erlangen, Germany). A phased-array head coil was used for head MRA and a neurovascular coil was used for neck MRA. Both ncTOF and cTOF head MRAs were performed in the axial plane with time of flight sequence -- repetition time (TR): 15.17-23; echo time (TE): 3.45-7; flip angle: 18°; slice: 1.4 mm; reconstruction interval: 0.7 mm; field of view (FOV): 160 mm; and matrix: 304 x 194 (reconstructed to 512 x 512). For the TOF technique, 0.1 mmol/kg of Magnevist was used as a contrast agent. CEMRA was performed with 0.2 mmol/kg of Magnevist, injected at the rate of 3-4 mL per second with a MR-compatible injector, and followed by a 20-cc saline flush. The antecubital vein was chosen for contrast administration. The parameters used with CEMRA of the neck included -- TR: ≤5.6; TE: ≤2.1; flip angle: 25° to 40°; slice: 1.5 to 3.0 mm; reconstruction interval: 0.8 to 1.5 mm; FOV: 200 mm for head and 350 mm for neck; and matrix: 288 x 288 to 416 x 344. CEMRA of the head was obtained in coronal and axial planes, and CEMRA of the neck in the coronal plane alone. In one case, dynamic CEMRA of the head was performed using time-resolved angiography with interleaved stochastic trajectories (TWIST, Siemens Medical Solutions USA, Inc., Malvern, PA). A bandwidth of 100-186 kHz with TOF techniques and 450-660 kHz with CEMRA techniques was used.

Three neuroradiologists independently assessed the maximum intensity projection (MIP) and source images for all MRA studies with the knowledge of location of coiled aneurysm and stent. Examiners noted the amount of flow signals through the stent, stent stenosis at the inlet and outlet zone, absent flow signals, and neck remnants versus residual or recurrent aneurysm. Stenosis was categorized as severe when the lumen at the stenotic site measured less than 50% diameter of the immediate proximal or distal segment of a stented artery. Intact flow signals proximal and distal to the stented segment of an artery with lack of flow signals in a portion of the stent was categorized as flow gap. In addition, examiners made a qualitative assessment of the superiority of a given technique based on visual perception and artifacts. Inter-rater agreement (kappa statistic) was assessed.

## Results

There were 36 males and six females in the study group. Ages ranged from 32 to 80 years. Embolization was performed using detachable platinum coils (Guglielmi Detachable Coils, Boston Scientific, Fremont, CA; and CERECYTE Microcoils, Micrus Endovascular, San Jose, CA). In total, 44 stents were deployed in 42 patients: 39 Neuroform stents (Boston Scientific, Natick, MA) and five Cordis Enterprise stents (Cordis Neurovascular, Miami Lakes, FL). Post stenting DSA was available in 32 cases. The time interval between MRA and DSA was less than three months in 22 cases, five to 15 months in nine cases and 27 months in one case. Non-contrast TOF MRA identified artifactual in-stent severe stenosis or flow void in 23 of 42 cases (55%). This feature was noted in 23 of 38 cases (60%) with cTOF MRA. Loss or reduction in flow signals was more often observed close to the junction of the stent and coiled aneurysm. DSA excluded stenosis in all 32 cases including those with artifactual stenosis in ncTOF and cTOF MRA (Table 1).

**Table 1:** Performance of various MRA techniques to identify flow across stent and residual aneurysm in 42 cases of stent-assisted coiling of intracranial aneurysm

	ncTOF MRA	cTOF MRA	CEMRA
Number of cases MRA done	42/42 (100%)	38/42 (90%)	8/42 (19%)
Artifactual severe stenosis or flow gap in stented segment	23/42 (55%)	23/38 (60%)	1/8 (13%)
Number of cases residual aneurysms identified	3	3	1
Number of cases neck remnants identified	3	3	None

**Figure 1:**
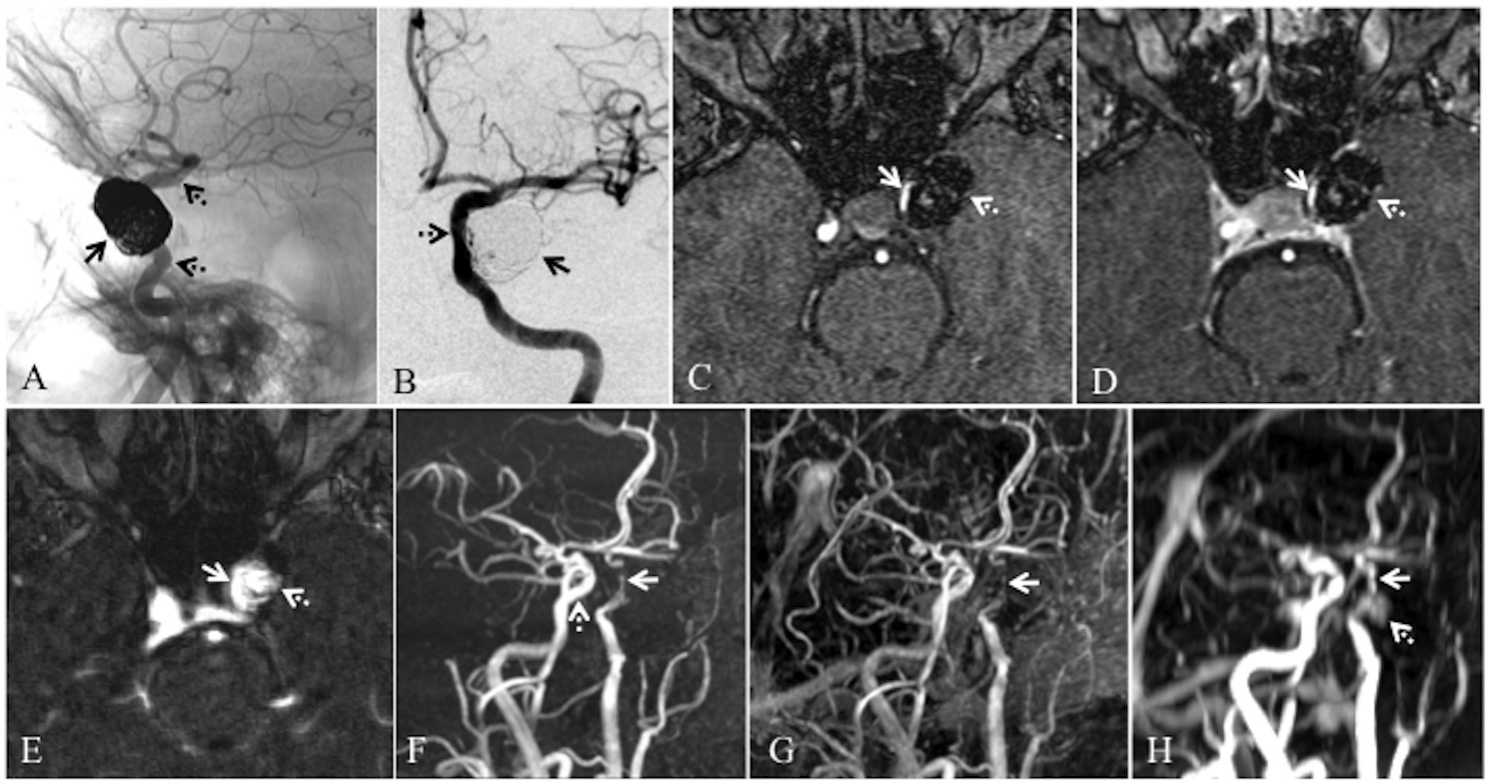
Left cavernous carotid aneurysm status post stent-assisted coil embolization and performance of three MRA techniques A: DSA selective left internal carotid injection unsubtracted image demonstrates the coil mass from embolized large left cavernous internal carotid artery (ICA) aneurysm (solid arrow). Proximal and distal markers of stent visible; deployed cavernous and proximal supraclinoid segments of left ICA visible (dotted arrows). B: Subtracted image of DSA anteroposterior view demonstrates fully patent lumen of ICA (dotted arrow). Minimal contrast filling is seen from residual aneurysm (solid arrow). C: ncTOF MRA raw data image shows flow signal in left cavernous ICA (solid arrow). Note the susceptibility from coil mass and very minimal flow signals inside the coil mass indicative of residual aneurysm (dotted arrow). D: cTOF MRA raw data image shows flow signal in left cavernous ICA (solid arrow) similar to ncTOF. Note the contrast enhancement of cavernous sinuses. The susceptibility from coil mass and flow signals inside from residual aneurysm appear identical to ncTOF (dotted arrow). E: Dynamic CEMRA raw data image shows better flow signals in left cavernous ICA (solid arrow) and residual aneurysm (dotted arrow). The residual aneurysm appears much larger. Note the reduction in susceptibility from coil mass compared to ncTOF and cTOF MRA raw data images. F: MIP image of ncTOF MRA in oblique sagittal view demonstrates loss of signals with severe stenosis like appearance through stent lumen in cavernous and proximal supraclinoid segments of left ICA. Note the focal complete loss of signals in proximal supraclinoid segment (solid arrow). There is no visualization of residual aneurysm. Intact flow is seen in right ICA (dotted arrow). G: MIP image of cTOF demonstrates similar appearance of loss of signals across the stent with no visualization of residual aneurysm (arrow). H: MIP image of dynamic CEMRA demonstrates better flow signals through the stent (solid arrow). Also, note the demonstration of residual aneurysm (dotted arrow).

Investigator I noted ncTOF was equal or superior to cTOF in 89% of cases, investigator II in 91% of cases and investigator III in 97% of cases. The kappa statistic revealed 0.87 agreement between investigator I and II, 0.81 between investigator I and III, and 0.83 between investigator II and III. No difference was noted between the investigators in identifying severe stenosis, flow gaps, residual aneurysm or recurrent aneurysm. Residual aneurysm was identified in three cases and neck remnants in three cases with ncTOF and cTOF MRA techniques. No difference was noted between ncTOF and cTOF in the demonstration of neck remnants or residual aneurysms. DSA was available in five cases with confirmation of two residual aneurysms and three neck remnants. CEMRA identified residual aneurysm in one case that also had ncTOF and cTOF MRA. In this case, 4D dynamic CEMRA demonstrated residual cavernous carotid artery aneurysm and flow through the stent much better than ncTOF and cTOF techniques. MIP images from both ncTOF and cTOF failed to demonstrate the residual aneurysm, which was evident only in the source images. However, the residual aneurysm was evident both in the MIP as well as source images of CEMRA (Figure 1).

In spite of using twice the slice thickness, CEMRA of the head and of the head and neck was rated superior by all three investigators. CEMRA showed a tendency to demonstrate superior in-stent flow, smoother margins and minimal or no stenosis in seven out of eight cases. One case of basilar artery stenting showed severe loss of flow signals with CEMRA and much worse signal loss with ncTOF and cTOF. This case had coil prolapse during stent-supported embolization, necessitating deployment of a second stent in a telescoping fashion to secure the prolapsed coil.

## Discussion

Magnetic resonance angiography is recommended as the technique of choice to monitor coiled intracranial aneurysms [11]. A subgroup of wide neck aneurysms are increasingly treated with stent-supported coil embolization, and there is a paucity of literature on MRA assessment of these cases [16-22].

Time of flight MRA has been used by few authors to specifically assess stent-assisted coil embolization [4,16,21-22]. Lovblad et al. [16] utilized a combination of ncTOF and cTOF with 1.5T magnetic resonance imaging (MRI) and assessed 19 cases of stent-assisted coil embolization. The authors made no comparison with DSA. They identified minimal artifacts with nitinol stents (Neuroform stent) and tram-track artifacts in nitinol stents with integrated radiopaque wire (Leo stent). In their study, stainless steel stents produced the most severe artifacts with complete loss of signals. Based on their results, they recommended cTOF MRA to assess stent-assisted coil embolization. Later, Wong et al. [4] assessed 15 cases of stent-assisted coil embolization with nitinol stents (Neuroform) using DSA and TOF MRA at 1.5T. They identified a statistical trend toward poor MRA visualization of the arterial lumen across stents. They provided no information on the utilization of intravenous contrast with TOF MRA. Recently, Cho et al. [21] reported better results with TOF MRA in the assessment of stent-assisted coil embolization of aneurysms. They assessed 20 cases of stent-assisted coiling of aneurysms and found high correlation between MRA and DSA in the identification of residual or recurrent aneurysms [21]. More recently, another group also reported better results with TOF MRA in the assessment of 40 cases of Enterprise stent-assisted coiling of aneurysms [22]. They found that MIP images, in combination with source images of MRA, reliably identified occlusion of coiled aneurysm. The authors reported in-stent stenosis of more than 33% in five cases, which was poorly visualized on both MIP and source images of TOF MRA. However, their analysis failed to note the status of flow signals across stents in TOF MRA [22].

In our study, ncTOF MRA showed severe in-stent stenosis or complete lack of flow signals across the stented segment in 55% of cases and cTOF MRA in 60% of cases. Our results fail to demonstrate the effectiveness of TOF MRA to assess stent-assisted coil embolization of aneurysms. Further, our results do not support the recommendation of contrast administration with TOF MRA to improve flow signals. Our results follow the trend established with TOF MRA assessment of coiled aneurysms without stents. In the assessment of coiled intracranial aneurysms without stent, very few studies recommend using cTOF, and many studies have reported the lack of an advantage for cTOF over ncTOF [7-10].

There are also few studies to date that have reported the role of CEMRA in evaluating intracranial stents with or without coiled aneurysms [17-20,23]. CEMRA can be acquired with first-pass circulation or dynamic technique. Dynamic contrast-enhanced MRA, which was used in one of our cases, is called by various names with different vendors including: time-resolved imaging of contrast kinetics (TRICKS), General Electric, Milwaukee, WI; TWIST, Siemens Medical Solutions USA, Inc., Malvern, PA; and 4D time-resolved MRA with keyhole (4D-TRAK), Philips Healthcare, Andover, MA. Masaryk et al.​[23] probably first reported the utility of CEMRA in the assessment of stent-assisted coil embolization. In a canine aneurysm model, the authors compared dynamic CEMRA technique against ncTOF and cTOF MRA and identified superior flow signals across nitinol stents with dynamic CEMRA [23]. In a direct comparison of first-pass CEMRA with 3D TOF in 10 cases of nitinol stent-supported coil embolization, Lubicz et al. [17] reported CEMRA was superior to TOF to assess aneurysm occlusion and parent artery patency. Azri et al., used first-pass CEMRA to assess 19 cases of stent-assisted coil embolization, and reported normal lumen or only mild stenosis across the stent in 13 of 19 cases. They identified moderate stenosis in four cases and severe stenosis in two cases. No comparison was made with TOF MRA [Al-Azri F, Santos M, Lesiuk H, Miller W, Goyal M, Lum C: Monitoring of intracranial aneurysms treated with “Neuroform” stent-assisted coiling using contrast-enhanced MR angiography. Poster from the 42nd Annual Congress, Canadian Neurological Sciences Federation. Edmonton, Alberta, Canada. June 2007.]. Takayama et al. [18] assessed five cases of stent-assisted coiling and found first-pass CEMRA at 1.5T MRI was superior to TOF MRA in its ability to demonstrate in-stent flow and residual/recurrent aneurysms. They noted no stenosis with CEMRA in all five cases and the stented lumen appeared similar to DSA. Further, they were able to assess the neck of treated aneurysms in all five cases [18]. Choi et al.,[19] using 3T MRI, found dynamic CEMRA was superior to TOF in the assessment of 26 cases of stent-assisted coil embolization. In their study, the concordance rate of TOF and dynamic CEMRA for evaluating the completeness of stent-assisted coil embolization was 57% and 79%, respectively. Agid et al. [20] analyzed first-pass CEMRA against DSA in 27 patients with 28 intracranial aneurysms. They found aneurysm remnants in 16 cases on CEMRA versus 13 cases on DSA. Further, they reported six cases of false stenosis and two cases of exaggerated stenosis on CEMRA. They found CEMRA to be an accurate technique to identify remnant aneurysms in stent-assisted coiling.

We used first-pass CEMRA in seven cases and dynamic CEMRA in one case. Although we obtained CEMRA as a head study alone or as a combination of head and neck, in all cases CEMRA demonstrated the stented lumen better than ncTOF and cTOF MRA techniques. CEMRA demonstrated good flow signals across the entire stented lumen in seven out of eight cases. In a single case, severe loss of in-stent flow signals were observed on CEMRA. Poor visualization of flow signals in this case occurred due to the deployment of double stents to jail a prolapsed coil. In the same case, ncTOF and cTOF MRA demonstrated much worse signal loss. The only dynamic CEMRA in our series demonstrated flow signals across the stented lumen in cavernous carotid artery almost similar to the contralateral normal side. Further, dynamic CEMRA revealed a large residual aneurysm, which was poorly visualized on both ncTOF and cTOF (Figure 1). We agree that our number of CEMRA cases is small, but they definitely reflect a trend of superior flow signals in stent and residual aneurysms. We need further studies in large patient populations to learn the utility of CEMRA in stent-assisted coil embolization.

Several important technical factors play a role in the signal loss from stents and coils. They include susceptibility effects, turbulence-induced intravoxel phase dispersion, saturation of signals from slow or stagnant flow and radiofrequency shielding effects [7-9,24]. Factors such as shortening TE, minimizing the pixel size by increasing the image matrix and reducing the imaging volume to target the stented segments should theoretically improve the signal loss from susceptibility and turbulence [24]. Also, increasing receiver bandwidth and decreasing field strength are expected to reduce susceptibility signal loss from coils and stents [9,19,25]. Radiofrequency shielding effects are thought to cause signal loss from cage-like effect and are mainly observed inside stent lumen [26]. We are unsure of the proportionate contribution of each of these technical factors to the signal loss with TOF MRA in stent-assisted coil embolization. Some reports indicate nitinol stents may be more susceptible to the radiofrequency-induced signal loss than susceptibility [25]. It is likely that the influence of various technical factors on magnetic resonance (MR) signals from inside stents and at the junction of stent and coil mass from aneurysm is dynamic and variable across magnets of equal strength and construction. This may explain the lack of correlation between our results and the results reported by others [16,21-22]. Examples of other conflicting reports between studies include the influence of voxel size, orientation of readout gradient to stent and orientation of stent to scan plane on susceptibility. It is well accepted that smaller voxel size should theoretically reduce susceptibility [24]. However, Seok et al. [25] reported that larger voxel size improved signals in stent lumen with TOF MRA. Some studies indicate parallel orientation of readout gradient to stent and static field improve MR signals [27]. Seok et al. [25] report perpendicular orientation of readout gradient to stent minimizes signal loss with TOF MRA. Stent composition and design also may influence signal loss [28]. Studies have reported more signal loss with Enterprise stent than with Neuroform [19,25,28]. Our number on Enterprise stents is too small to make a valid comment on the status of inferior flow signals versus Neuroform stent. Agid et al. reported focal pseudostenosis near the marker bands of stents especially when the distal tip of stent was located in smaller branches measuring less than 2 mm in diameter. The authors called it "marker band effect" [20]. We also identified similar signal loss in some cases in our series. Some of the potential advantages with CEMRA over TOF MRA in the assessment of stent-assisted coil embolization include less susceptibility artifacts from short TE as well as larger bandwidth and superior background suppression. As our results reflect, CEMRA may be better suited to assess stent-assisted coil embolization of aneurysms, with demonstration of superior flow signals across stent and residual or recurrent aneurysms. Further, it may minimize false-positive aneurysms from intraluminal thrombus with superior suppression of background signals.

The important pitfalls of our study include relatively small patient population, retrospective nature of the study and lack of DSA in one-third of cases. Nevertheless, ours is one of the largest series comparing ncTOF to cTOF in the assessment of stent-supported coiling of intracranial aneurysms. Our results on TOF MRA are in conflict with the results obtained by others [16,21-22]. We require prospective analysis of a large patient population with TOF MRA, CEMRA and dynamic CEMRA to identify the best technique to assess stent-assisted coil embolization of intracranial aneurysms. Further, it will be interesting to know whether magnets of equal strength and construction induce dynamic and variable influence on MR signal loss inside the lumen of stents with identical composition and construction.

## Conclusions

In the assessment of stent-assisted coil embolization of intracranial aneurysms, both ncTOF and cTOF MRA show similar results with signal loss from stents in more than half the cases. Contrast administration does not provide any additional benefit to TOF MRA to improve flow signals inside stent and in residual or recurrent aneurysm. CEMRA tends to show better flow signals inside stent and in residual aneurysms. The use of a short TE and larger bandwidth inherent in CEMRA, particularly dynamic contrast enhanced MRA, may be promising to assess stent-assisted coil embolization. It is possible MR signals in stent lumen from the same patient are dynamic and variable across magnets of equal strength and construction. Our study emphasizes the need for a prospective analysis of a large patient population comparing the various MRA techniques in the assessment of stent-assisted coil embolization.
